# CT, MRI, and F-18 FDG PET for the detection of non-small-cell lung cancer (NSCLC): A protocol for a network meta-analysis of diagnostic test accuracy

**DOI:** 10.1097/MD.0000000000012387

**Published:** 2018-09-21

**Authors:** Yi Zhang, Jinman Ni, Kongyuan Wei, Jinhui Tian, Shaobo Sun

**Affiliations:** a School of Basic Medical Sciences, Lanzhou University; b Department of General Surgery, First Affiliated Hospital of Lanzhou University; c Evidence-based Medicine Center, School of Basic Medical Sciences; d Gansu university of Chinese Medicine, Lanzhou University, Lanzhou, China.

**Keywords:** CT, F-18 FDG PET, MRI, network meta-analysis, non-small-cell lung cancer, sensitivity, specificity

## Abstract

**Background:**

Non-small-cell lung cancer (NSCLC) is a rare cancer in lung carcinomas and has been widely known as a difficult curable disease among all the tumors. However, early detection of malignant potential in patients with NSCLC has still been a huge challenge all around the world. CT, MRI, and F-18 FDG PET are all considered as good tests for diagnosing malignant NSCLC efficiently, but no recommended suggestion presents that which test among the 3 is the prior one in diagnose. We perform this study through network meta-analysis method, and to rank these tests using a superiority index.

**Methods and analysis:**

PubMed, Embase.com, and the Cochrane Central Register of Controlled Trials (CENTRAL) will be searched from their inception to March 2018. We will include diagnostic tests which assessed the accuracy of CT, MRI, and F-18 FDG PET for diagnosing NSCLC. The risk of bias for each study will be independently assessed as low, moderate, or high using criteria adapted from Quality Assessment of Diagnostic Accuracy Studies 2 (QUADAS-2). Network meta-analysis will be performed using STATA 12.0 and R 3.4.1 software. The competing diagnostic tests will be ranked by a superiority index.

**Results:**

This study is ongoing, and will be submitted to a peer-reviewed journal for publication.

**Conclusion:**

This study will provide systematically suggestions to select different diagnostic measures for detecting the early NSCLC.

**Ethics and dissemination:**

Ethical approval and patient consent are not required since this study is a network meta-analysis based on published studies. The results of this network meta-analysis will be submitted to a peer-reviewed journal for publication.

**PROSPERO registration number:**

PROSPEROCRD42018094542.

## 1 Introduction

Non-small-cell lung cancer (NSCLC) is a malignant cancer ranking the most common causes to death in Western countries,^[1]^ although there are advanced therapies in the world now, the early detection of NSCLC has still been a tough problem all around the world.^[2]^ According to the recently published guideline^[3]^ for the management to the patients with NSCLC, diagnose of early NSCLC is considered to be the effective solution to reduce the mortality of NSCLC.

In the past few years, lung cancer incidence rates decreased by 1.9% per year in men and by 1.2% per year in women. In 2014, an estimated 224,210 new cases and 159,260 deaths of lung cancer are expected in the United States.^[4]^ Fortunately, there have been chemotherapies for the patients with NSCLC while only part of them are resistant to the treatment, so it is also mandatory to predict the response to treatment for the malignant NSCLC. However, preoperative evaluation of malignant NSCLC remains difficult for most NSCLC are hard to assess the stage of malignant potential.^[5]^

As we all know, CT, MRI, and F-18 fluorodeoxyglucose (FDG) positron emission tomography (PET) or positron emission tomography or computed tomography (PET/CT) were proved to useful for diagnosing tumor staging in different cancers.^[6]^ CT is a common imaging modality for lots of tumors, besides, for the patients with GISTs, it is also functional to predict the response of treatment^[7]^.Likewise, MRI is considered efficient to detect malignant NSCLC at very early stage, which will be helpful for the patients with NSCLC receiving chemotherapy treatment.^[8]^Moreover, F-18 FDG PET or PET/CT has been proved to present high diagnostic accuracy for the patients with malignant potential.^[9,10]^

To the best of our knowledge, this meta-analysis might be the first study to compare the efficacy of all the 3 approaches to diagnose the malignant potential of NSCLC at quite early stage, there was no recommended early detection for the malignant potential of NSCLC, so our study aims to compare the diagnostic accuracy of CT, MRI, and F-18 FDG PET or PET/CT in order to provide better suggestions for the clinic and patients with NSCLC though network meta-analysis method and to rank these tests using superiority index.

## 2 Method

### 2.1 Eligibility criteria

#### 2.1.1 Type of study

Eligible studies are as follows: index tests include either CT, MRI, and F-18 FDG PET or PET/CT or combinations; at least 2 index tests per study, one of them being CT; report or provide sufficient information to allow us to calculate the true positive (TP), false positive (FP), true negative (TN), and false negative (FN) values; case-control, cross-sectional, or cohort designs; there will be no limitations on language of publication, year of publication.

#### 2.1.2 Patients

We will include studies that contain patients performed on CT or MRI or F-18 FDG PET or PET/CT to predict malignant potential of NSCLC. We will exclude studies that provide no sufficient data of diagnostic accuracy. We will put no limitations in age, gender, and nations.

#### 2.1.3 Index tests

We will regard CT, MRI, and F-18 FDG PET or PET/CT as index tests because these tests are usually used to predict malignant potential of NSCLC. Study inclusion based on the diagnostic criteria that were used will not be limited while study inclusion based on the quality of CT, MRI, and F-18 FDG PET or PET/CT will be limited.

#### 2.1.4 Reference standards

Definitive histopathology following surgery will be considered as primary reference standard and the clinical follow-up after treatment will be the complementary reference standard.

#### 2.1.5 Outcomes

The primary outcomes are sensitivity, specificity, and diagnostic odds ratios (DOR).The second outcomes are relative sensitivity, relative specificity, and relative diagnostic odds ratio.

### 2.2 Information sources

PubMed, Embase, and the Cochrane Central Register of Controlled Trials (CENTRAL) will be searched until April 2018. The search strategies will be conducted by ZY and NJM who are experienced information specialists. The references of relevant systematic reviews/meta-analyses will be searched to identify additional potential studies.

### 2.3 Search strategy

Search strategy of PubMed was as follows:

#1 ((((((“NSCLC”[MeSH Terms]) OR “nsclc”[Title/Abstract]) OR “Non-Small-Cell Lung Cancer”[Title/Abstract])OR “non-small-cell lung cancer”[Title/Abstract]) OR “non-small-cell lung carcinoma”[Title/Abstract]) OR “Non-Small-Lung Cancers”[Title/Abstract])
#2 (((((CT [MeSH Terms]) OR ct [Title/Abstract]) OR computer tomography [Title/Abstract]) OR Computed Tomography [Title/Abstract]) OR Computer tomography [Title/Abstract])))
#3 (((((“MRI”[MeSH Terms]) OR“Magnetic Resonance Imaging”[Title/Abstract]) OR “Nuclear Magnetic Resonance Imaging”[Title/Abstract]) OR “nuclear magnetic resonance imaging”[Title/Abstract]) OR “Nuclear Magnetic Resonance Imagings ”[Title/Abstract])
#4 (((((PET/CT [MeSH Terms]) OR Positron Emission Computed Tomography [Title/Abstract]) OR PET [Title/Abstract]) OR F-18 FDG [Title/Abstract]) OR 18F-FDG PET-CT [Title/Abstract])))
#5 #2 AND #3
#6 #2 AND #4
#7 #5OR #6
#8 #1 AND #7

### 2.4 Study selection and data extraction

Microsoft Excel 2013 (Microsoft Corp, Redmond, WA, www.microsoft.com) will be used to collect data, which include eligible studies characteristics (e.g., name of first author, year of publication, country in which the study was conducted, gold standard, index tests), patients characteristics (male, mean age, sample, method, cutoff level, risk factors of NSCLC), and outcomes (SEN, SPE, TP, FP, FN,TN).

Study selection and data extraction will be conducted by one reviewer (ZY), and will be checked by other reviewers (NJM and WKY). If there exist conflicts, conflicts will be resolved by a third reviewer (SSB).

### 2.5 Quality evaluation

The risk of bias will be independently evaluated by 2 reviewers (ZY and NJM) for each study as low, moderate, or high using criteria adapted from the Quality Assessment of Diagnostic Accuracy Studies 2 (QUADAS-2).^[11]^ If there exist conflicts, conflicts will be resolved by discussion.

### 2.6 Geometry of the network

We will network meta-analyses pooled data using R software version 3.4.1. In network plots, the size of the nodes is proportional to the number of studies evaluating a test, and thickness of the lines between the nodes is proportional to the number of direct comparisons between tests. The network is connected because there is at least one study evaluating a given test together with at least one of the other remaining tests.^[12]^ A loop connecting 3 tests indicates that there is at least one study comparing the 3 targeted tests simultaneously.^[13]^

### 2.7 Network meta-analysis

#### 2.7.1 Pairwise meta-analyses

We will perform pairwise meta-analyses for pooled sensitivity (SEN), specificity (SPE), positive likelihood ratio (PLR), negative likelihood ratio (NLR), diagnostic odds ratio (DOR), and area under the summary receiver operating characteristic curve (AUSROC) using bivariate mixed-effects regression modeling with STATA version 12.0 (Stata, College Station, TX). The between-study variance will be calculated var logitSEN and logitSPE.^[14]^ The proportion of heterogeneity according to the threshold effect among the included studies will be calculated by the squared correlation coefficient estimated from the between-study covariance variable in the bivariate model.^[15]^ The heterogeneity between each study will be estimated using the *Q* value and the inconsistency index (*I*^2^) test, and the values of 25%, 50%, and 75% for the *I*^2^ will be indicative of low, moderate, and high statistical heterogeneity, respectively.^[16]^

Subgroup analyses for each will be conducted on the basis of the country in which the study was conducted, cutoff level, and risk of bias.

Deek's funnel plot will be carried out to evaluate the potential publication bias when there are more than 10 studies available for an index test.^[17]^

#### 2.7.2 Indirect comparisons between competing diagnostic tests

We will calculate relative diagnostic outcomes between index tests by ANOVA model in R software version 3.4.1,^[12]^ including relative sensitivity (RSEN), relative specificity (RSPE), and relative diagnostic odds ratio (RDOR).

#### 2.7.3 Ranking of competing diagnostic tests

Some researchers regard DOR as an indicator of ranking of competing diagnostic tests^[18]^ while the measure might not distinguish between tests with high sensitivity but low specificity or vice-versa. Besides, the superiority index introduced by Deutsch et al^[19]^ provides more weight to tests performing relatively well on both diagnostic accuracy measures and less weight on tests performing poorly on both diagnostic measures or tests performing better on one measure but poorly on the other.^[12]^The superiority index ranges from 0 to ∞, and tends toward ∞ and 0 as the number of tests to which the target test is superior and inferior increases, respectively, and superiority index tending to 1 indicates that the tests are equal.^[12]^

### 2.8 Assessment of reporting bias

Due to lack of sensitivity tests in diagnostic test accuracy reviews and in determination of publication bias, we will not investigate reporting bias.^[20]^

## 3 Discussion

Lung cancer has been considered to be the most dangerous cause among all the carcinomas leading to deaths no matter in developed countries or developing nations.^[21]^ The early detection of NSCLC plays a necessary role in treating the disease at quite early stage, on the other hand, there are kinds of approaches diagnosing NSCLC in clinic. However, until now, rare suggestions are offered to choose an effective and safe measure to assess the malignant potential of NSCLC in order to improve the quality of living conditions of patients with NSCLC.

To best of our knowledge, this will be the first network meta-analysis to compare the efficacy and diagnostic accuracy among all the 3 measures diagnosing NSCLC at a definitely early stage. Through analyzing all the included studies, we will present a beneficial tool to diagnose NSCLC and make a priority sequence for all the 3 ways. We hope we might provide robust and elaborate evidences for the clinical practise in large scale.

## Author contributions

ZY, NJM, and WKY plan and design the research; ZY, NJM and TJH tested the feasibility of the study; ZY, WKY and SSB provided methodological advice, polished and revised the manuscript; NJM and SSB wrote the manuscript; all authors approved the final version of the manuscript.

**Data curation:** Jinman Ni, Jinhui Tian.

**Methodology:** Jinman Ni.

**Software:** Jinhui Tian.

**Writing – original draft:** Yi Zhang, Kongyuan Wei.

**Writing – review & editing:** Shaobo Sun.
